# Supplementary material for: Sedentary Duration and Systemic Health Burden: Nonlinear Associations with Muscle, Fat, and Vascular Phenotypes in a US Population-Based Study
Source: Healthcare (Basel). 2025 Sep 16;13(18):2309. doi: 10.3390/healthcare13182309 (PMC12469714; doi:10.3390/healthcare13182309)
Supplement: Supplementary file 1 [file healthcare-13-02309-s001.zip › healthcare-3752733-supplementary.pdf]

Table S1: Baseline Characteristics of Participants

Characteristic	N[1]	Overall	Q1	Q2	Q3	p-value[2]
Sex	13,521					0.057
Male		6,801	2,459	1,903	2,4	
Female		6,720	2,237	2,164	2,3	
Age(years)	13,521	33.42±	37.13±	32.80±	29.	<0.001
Race	13,521					<0.001
Mexican		2,330	970 (15%)	726 (11%)	634	
Other		1,434	618 (9.6%)	392 (6.9%)	424	
Non-Hispanic		4,357	1,496	1,333	1,5	
Non-Hispanic		2,838	855 (10%)	857 (11%)	1,1	
Non-Hispanic		1,869	561 (5.0%)	585 (6.3%)	723	
Other Race -		693 (4.0%)	196 (4.0%)	174 (3.8%)	323	
Education Level	13,521					<0.001
Less than 9th		1,070	452 (6.6%)	281 (4.9%)	337	
9-11th grade		1,701	702 (12%)	488 (9.7%)	511	
High school		2,860	1,154	831 (20%)	875	
Some college		4,235	1,479	1,306	1,4	
College		3,655	909 (23%)	1,161	1,5	
Marital Status	13,521					<0.001
Married		5,089	2,239	1,441	1,4	
Widowed		496 (2.6%)	90 (1.5%)	128 (2.3%)	278	
Divorced		1,027	352 (7.3%)	303 (8.6%)	372	
Separated		685 (4.0%)	216 (4.2%)	182 (3.8%)	287	

Never		4,485	1,237	1,446	1,8	
Living with		1,739	562 (11%)	567 (13%)	610	
Alcohol	13,521	3.44±	3.31±	3.31±	3.7	<0.001
Sleep	13,521	7.32±	7.29±	7.35±	7.3	0.710
Cigarettes/Day	13,521	11.73±	12.05±	11.70±	11.	<0.001
General Health	13,521					0.082
Excellent		1,604	524 (12%)	496 (12%)	584	
Very good		4,152	1,322	1,294	1,5	
Good		5,455	1,966	1,596	1,8	
Fair		1,951	789 (14%)	564 (12%)	598	
Poor		359 (2.1%)	95 (1.7%)	117 (2.1%)	147	
Like to Weigh	13,521					<0.001
More		1,884	557 (11%)	569 (12%)	758	
Less		7,217	2,639	2,160	2,4	
Same		4,189	1,495	1,276	1,4	
Don't know		231 (1.0%)	5 (<0.1%)	62 (1.0%)	164	
Systolic Blood	13,521	115.04±	117.02±	114.73±	113	<0.001
Diastolic Blood	13,521	68.71±	70.54±	68.53±	66.	<0.001
Total Area (cm^2)	13,521	2,048.84±	2,099.04±	2,053.46±	1,9	<0.001
Total Bone	13,521	2,250.82±	2,353.40±	2,258.69±	2,1	<0.001
Total Bone	13,521	1.09±	1.11±	1.09±	1.0	<0.001
Trunk Percent	13,521	30.35±	30.50±	30.18±	30.	0.308
Total Percent Fat	13,521	31.95±	31.59±	31.83±	32.	0.004
Left Arm Lean	13,521	2.86±	3.03±	2.86±	2.6	<0.001

Left Leg Lean	13,521	7.89±	8.10±	7.94±	7.5	<0.001
Right Arm Lean	13,521	3.04±	3.22±	3.04±	2.8	<0.001
Trunk Fat (g)	13,521	11,817.35±	12,153.13±	11,897.72±	11,	<0.001
Left Arm Fat (g)	13,521	1,536.40±	1,550.65±	1,543.02±	1,5	0.007
Right Arm Fat	13,521	1,545.25±	1,561.19±	1,550.04±	1,5	0.004
Left Leg Fat (g)	13,521	4,551.90±	4,493.36±	4,571.26±	4,6	0.558
Right Leg Fat (g)	13,521	4,667.38±	4,613.05±	4,688.00±	4,7	0.709
Right Leg Lean	13,521	8.10±	8.32±	8.16±	7.7	<0.001
BMI (kg/m^2)	13,521	27.23±	27.77±	27.25±	26.	<0.001
Waist	13,521	92.88±	94.72±	92.87±	90.	<0.001
SI	13,521	0.81±	0.83±	0.82±	0.7	<0.001
FDI	13,521	0.95±	1.00±	0.94±	0.8	<0.001
Pulse Pressure	13,521	46.33±	46.48±	46.20±	46.	0.701
PPI	13,521	0.40±	0.40±	0.40±	0.4	<0.001

[1]N not Missing (unweighted)
[2]Pearson's X^2: Rao & Scott adjustment; Design-based KruskalWallis test
